# A multicenter cross-sectional survey on the status and influencing factors of health communication competence among primary healthcare workers

**DOI:** 10.3389/fpubh.2025.1713479

**Published:** 2025-12-15

**Authors:** Xian Zhao, Jialin Zhao, Zhixia Shi, Tingting Li, Lili Zhao, Rou Li, Ling Li

**Affiliations:** 1 Institute of Health Education, Zibo Center for Disease Control and Prevention, Zibo, Shandong, China; 2 Department of Maternal and Child Health, Zichuan District Hongshan Health Center, Zibo, Shandong, China; 3 Department of Food Nutrition and Student Health, Zibo City Zhangdian District Disease Control and Prevention Center, Zibo, Shandong, China

**Keywords:** primary healthcare workers, health communication, health literacy, influencing factors, cross-sectional study

## Abstract

**Objective:**

To evaluate the current status and influencing factors of health communication competence among primary healthcare workers, providing evidence for improving the quality of primary health promotion services.

**Methods:**

A cross-sectional survey was conducted in August 2025 among 5,449 primary healthcare workers in Zibo City, Shandong Province, using multi-stage stratified random sampling. A self-designed questionnaire assessed four dimensions: health literacy mastery, knowledge acquisition ability, communication practice behavior, and policy cognition level. Statistical analyses included *t*-tests, ANOVA, multiple linear regression, and structural equation modeling (SEM).

**Results:**

The overall competence score was 69.45 ± 16.23, indicating a moderate level. Scores for health literacy mastery were highest, while communication practice behavior was lowest, showing a knowledge–practice gap. Education level, professional title, self-rated health status, and institution type were positive predictors, whereas occupational stress negatively affected competence (*P* < 0.001). The final regression model explained 42.1% of variance (*R*^2^ = 0.421). SEM confirmed the direct and indirect effects of these factors, with good model fit (*χ*^2^/df = 2.34, RMSEA = 0.045, CFI = 0.96, TLI = 0.95).

**Conclusion:**

Health communication competence among primary healthcare workers remains moderate and unevenly distributed. Targeted interventions focusing on education, professional development, health promotion training, and stress reduction are essential to enhance competence and improve the effectiveness of primary health communication.

## 1 Introduction

Health communication is recognized as a fundamental driver for achieving the “Healthy China 2030” strategic goals, critically bridging the gap between medical knowledge and public action to enhance population-wide health literacy and bolster disease prevention efforts (1). Within this framework, primary healthcare workers (PHCWs) act as the first line of defense, serving as health gatekeepers in their communities. Their capacity to effectively communicate health information is paramount, directly influencing the dissemination efficacy of health knowledge and the subsequent adoption of healthy behaviors among the public (2, 3). The World Health Organization has consistently underscored that strengthening health promotion competencies, including communication skills, within the primary health workforce is indispensable for achieving universal health coverage (4).

Health communication competence is conceptualized as a multidimensional construct comprising four interrelated components: (1) health literacy mastery, representing the ability to comprehend, evaluate, and apply health information in professional and community settings; (2) knowledge acquisition ability, referring to the continuous learning, updating, and integration of professional and public health knowledge through formal and informal channels; (3) communication practice behavior, denoting the application of communication strategies and interpersonal skills in patient counseling, health education, and community outreach; and (4) policy cognition level, reflecting awareness, understanding, and engagement with national health policies and health promotion programs. These four dimensions are theoretically grounded in Nutbeam’s health literacy framework and the Knowledge–Attitude–Practice (KAP) model, both of which emphasize the transformation from knowledge acquisition to behavioral practice and policy-level cognition (5). This integrated conceptualization allows for a more comprehensive understanding of PHCWs’ competencies in the context of preventive health service delivery.

Despite the growing policy emphasis on health education within primary care, the communication competence of PHCWs remains heterogeneous and generally suboptimal. Prior studies indicate that disparities in educational background, occupational stress, institutional resources, and self-rated health status—key independent variables (IVs) in this study—may significantly influence communication capacity (6, 7). However, existing evidence is fragmented and often lacks multidimensional evaluation frameworks or large-scale empirical validation. Consequently, current understanding of the determinants of PHCWs’ health communication competence remains limited, underscoring the need for integrated analytical models and evidence-based strategies for improvement.

To address these gaps, this study developed a holistic evaluation framework that assesses four key dimensions: health literacy mastery, knowledge acquisition ability, communication practice behavior, and policy cognition level. Through a multi-center, cross-sectional survey, the study aim to: (i) provide a comprehensive assessment of the current status of health communication competence among PHCWs; (ii) elucidate the complex interplay of influencing factors spanning individual, occupational, and health-related domains; and (iii) offer evidence-based recommendations to inform policy and practice aimed at optimizing the health promotion service system.

## 2 Methods

### 2.1 Study population and sampling

A multi-center, cross-sectional survey was conducted in Zibo City, Shandong Province, in August 2025. Participants were selected using a multi-stage stratified random sampling method. Primary healthcare institutions were first stratified by type (community health service centers/stations, township health centers, village clinics, outpatient departments, and private clinics). Within each stratum, institutions were randomly selected using a random number table.

Eligibility criteria for participants included: (1) employment at a primary healthcare institution for at least 6 months; (2) possession of a valid professional practice certificate; (3) direct involvement in clinical work (e.g., medical, nursing, pharmaceutical, or technical services); and (4) voluntary participation. Exclusion criteria were: (1) administrative or logistical staff; (2) individuals in training or internship positions; and (3) those on leave or away for training during the survey period.

The sample size was calculated based on a preliminary survey indicating an estimated “good” or “excellent” health communication competence prevalence of 70% (*P* = 0.70). Using the formula for cross-sectional studies, *n* = *Z*^2^*P*(1 − *P*)/*d*^2^ (where *Z* = 1.96 for a 95% confidence level and *d* = 0.02 for a 2% margin of error), a minimum sample size of 2017 was required. After considering a design effect of 1.5 due to the multi-center structure and a 20% anticipated rate of invalid responses, the target sample size was set at 5000.

A proportionate stratified random sampling approach was applied to ensure representativeness across districts and institution types. The sampling frame was compiled in collaboration with the Zibo Municipal Health Commission, which provided official rosters of eligible primary healthcare workers from each institution type. Within each stratum, participants were randomly selected using a random-number table. Slight oversampling was performed to compensate for potential non-response, resulting in 5684 questionnaires collected and 5,449 valid responses after data cleaning, yielding an effective response rate of 95.87%.

### 2.2 Research instruments

#### 2.2.1 Demographic characteristics questionnaire

A structured questionnaire was used to collect demographic and occupational data, including gender, age, educational level, professional category, technical title, years of work experience, type of institution, size of population served, and average monthly income. This ensured standardized and comparable data collection.

#### 2.2.2 Health communication competence assessment scale

Health communication competence was assessed using a self-designed scale adapted from the Health Communication Efficacy Scale and the Health Information Dissemination Behavior Scale. The instrument comprised three primary domains (proactive communication, passive communication, and academic communication) with 15 secondary indicators. Items were rated on a 5-point Likert scale (1 = never, 5 = always). Domain scores were weighted and converted to a standardized score ranging from 0 to 100.

#### 2.2.3 Health knowledge acquisition and learning behavior tool

This section, developed with reference to knowledge management behavior scales, assessed various learning pathways: formal learning (frequency of participation in continuing medical education, pursuit of advanced degrees), self-directed learning (frequency of consulting professional books, searching Chinese/English literature databases, browsing professional medical websites, using mobile medical apps), collaborative learning (frequency of peer communication, participation in multidisciplinary team discussions), and problem-solving strategies (evidence-based practice, consulting clinical guidelines, seeking expert consultation).

#### 2.2.4 Occupational stress measurement scale

Occupational stress was measured using the Chinese version of the Occupational Stress Inventory-Revised (OSI-R). This study focused on six dimensions from the Occupational Roles Questionnaire (ORQ): role overload, role insufficiency, role ambiguity, role boundary, responsibility, and physical environment; and the social support dimension from the Personal Resources Questionnaire (PRQ). Each dimension consisted of 5 items scored on a 5-point scale (1 = never, 5 = always). Dimension scores were calculated as the mean of item scores multiplied by 10.

#### 2.2.5 Chinese citizen health literacy questionnaire

Participants’ health literacy was evaluated using a questionnaire based on the “National Resident Health Literacy Monitoring Questionnaire,” adapted for healthcare professionals. It assessed three core areas (basic health knowledge and concepts, healthy lifestyle and behaviors, and basic health skills) and six specific health problem domains (safety and first aid, scientific health concepts, health information literacy, basic medical care, infectious disease prevention, and chronic disease prevention). The questionnaire contained 17 items (2 true/false, 10 single-choice, 5 multiple-choice). True/false and single-choice items scored 1 point for correct answers; multiple-choice items scored 2 points for fully correct answers. The total possible score was 22. A total score ≥18 (80% of the maximum) indicated adequate health literacy. Scores for the three core areas and six problem domains were presented as percentages.

#### 2.2.6 Health policy cognition and attitude assessment scale

Based on the Knowledge-Attitude-Practice (KAP) model, this scale assessed policy awareness (correct rate scoring), policy understanding depth (correct rate scoring), policy participation (frequency quantification), policy attitude, policy implementation evaluation, and health communication willingness (the latter three used 7-point Likert scales).

#### 2.2.7 Instrument reliability and validity

To ensure the measurement quality of the self-designed and adapted instruments used in this study, a pilot test was conducted among 120 primary healthcare workers from three community health centers and two township health institutions in Zibo City prior to the formal survey. The reliability and validity of all scales were evaluated.

The internal consistency reliability (Cronbach’s *α*) of the health communication competence Assessment Scale, Health Knowledge Acquisition and Learning Behavior Tool, and Health Policy Cognition and Attitude Assessment Scale were 0.912, 0.886, and 0.894, respectively, indicating high reliability. The Cronbach’s *α* values of all subdimensions ranged from 0.812 to 0.923. Test–retest reliability, assessed after a two-week interval among 60 participants, ranged from 0.831 to 0.905.

Construct validity was examined using exploratory and confirmatory factor analyses. The Kaiser–Meyer–Olkin (KMO) values of the three core instruments were 0.906, 0.884, and 0.873, respectively, and Bartlett’s test of sphericity was significant (*P* < 0.001), supporting factorability. The cumulative variance explained by the extracted factors exceeded 68% for all instruments, and standardized factor loadings of all retained items were greater than 0.60. These results indicate satisfactory construct validity. Content validity was further confirmed by a panel of five experts in health communication, health education, and psychometrics, yielding a scale-level content validity index (S-CVI) of 0.92.

Collectively, these results demonstrate that the instruments used in this study possessed good reliability and validity for assessing health communication competence and its related constructs among primary healthcare workers.

### 2.3 Quality control

The survey was administered online via the Questionnaire Star platform. Careful attention was paid to questionnaire design, including instructions, question types, and logical skip patterns. A double-check procedure was implemented by two researchers to ensure accurate distribution and collection. To verify data authenticity, 5% of respondents were randomly selected for telephone follow-up. A total of 5,449 valid questionnaires were collected.

To minimize potential response bias, all questionnaires were completed anonymously through an online platform. Participants were informed that their responses would be used solely for academic research, and no identifying information was collected. Instructions emphasized voluntary participation and honesty to reduce possible social desirability bias, especially regarding self-perceived competence and occupational stress.

### 2.4 Statistical analysis

Data analyses were conducted using SPSS version 20.0 (IBM Corp., Armonk, NY, USA) for descriptive statistics, group comparisons, and regression analyses. Continuous variables were summarized as mean ± standard deviation (SD). Normality and homogeneity of variance were examined before group comparisons. For data that were normally distributed with equal variances, *t*-tests or one-way ANOVA were applied; otherwise, Mann–Whitney U and Kruskal–Wallis H tests were used. Categorical variables were expressed as frequencies and percentages and compared using the chi-square (*χ*^2^) test.

Correlations between knowledge-acquisition pathways and health-communication competence were evaluated using Pearson’s correlation for normally distributed variables and Spearman’s rank correlation for non-normal variables. The influence of occupational stress on health-communication competence was analyzed through linear-regression modeling after confirming residual normality and homoscedasticity; regression coefficients (*β*) and 95% confidence intervals (CIs) were reported. The association between health literacy [dichotomized as adequate (score ≥ 18) vs. inadequate (score < 18)] and communication competence was examined using binary logistic regression to estimate odds ratios (ORs) and 95% CIs.

Multivariable relationships were further explored through multiple linear-regression analysis. Stepwise regression was implemented for variable selection to reduce overfitting and omission of significant predictors; the decision criteria and model diagnostics were verified. Structural-equation modeling (SEM) was then performed to investigate the complex inter-relations among influencing factors. Model-fit adequacy was confirmed using the *χ*^2^/df ratio, Root Mean Square Error of Approximation (RMSEA), Comparative Fit Index (CFI), and Tucker–Lewis Index (TLI).

Prior to regression analyses, multicollinearity among independent variables (e.g., education level, professional title, work experience) was assessed by variance-inflation factors (VIFs), all <5, indicating acceptable tolerance. Although stepwise regression can be prone to overfitting, sensitivity checks produced consistent results. No correction for multiple comparisons was applied in univariate analyses; therefore, the potential inflation of Type I error was acknowledged in result interpretation. All statistical tests were two-tailed, and *P* < 0.05 was considered statistically significant. The complete dataset is available from the corresponding author upon reasonable request.

## 3 Results

### 3.1 Comprehensive evaluation of health communication competence

A total of 5,449 valid questionnaires were collected, and the overall response rate was 96.1%. Among the respondents, 1,750 (32.1%) were male and 3,699 (67.9%) were female. The average age was 38.64 ± 8.57 years. This study established a health communication competence evaluation system encompassing four dimensions: mastery of health literacy, knowledge acquisition ability, communication practice behavior, and policy cognition level. Based on the questionnaire data, the 5,449 primary healthcare workers were categorized according to their total capacity scores into a low-level group (≤60 points, *n* = 1,634, 30.00%), a medium-level group (61–80 points, *n* = 2,723, 49.97%), and a high-level group (≥81 points, *n* = 1,092, 20.04%). The comprehensive health communication competence score was 69.45 ± 16.23 points. Among the dimensions, the Health Literacy Mastery dimension scored the highest (78.32 ± 12.45 points), while the Communication Practice Behavior dimension scored relatively lower (62.78 ± 18.67 points), as detailed in Table 1.

**Table 1:** Comprehensive evaluation results of health communication competence among primary healthcare workers.

Evaluation dimension	Full score	Score	Excellent [*n* (%)]	Good [*n* (%)]	Average [*n* (%)]	Poor [*n* (%)]
Health literacy mastery	100	78.32 ± 12.45	1,456 (26.73)	2,389 (43.84)	1,378 (25.29)	226 (4.15)
Knowledge acquisition ability	100	71.89 ± 15.67	978 (17.95)	2,167 (39.78)	1834 (33.66)	470 (8.62)
Communication practice	100	62.78 ± 18.67	654 (12.00)	1789 (32.84)	2,234 (41.02)	772 (14.17)
Policy cognition level	100	75.56 ± 14.23	1,234 (22.65)	2,567 (47.11)	1,423 (26.12)	225 (4.13)
Comprehensive score	100	69.45 ± 16.23	1,092 (20.04)	2,723 (49.97)	1,634 (30.00)	–

### 3.2 Health literacy mastery level of primary healthcare workers

The overall mastery of health literacy among primary healthcare workers was generally good, with notable variation across domains. High accuracy was observed for basic concepts and infectious disease prevention, whereas prenatal care cognition showed relatively lower accuracy. Complex skill proficiency was strongest for cardiopulmonary resuscitation (CPR) and public health service cognition. Detailed distributions of correct response rates and skill proficiency levels are shown in Table 2.

**Table 2:** Health literacy mastery and skill proficiency among primary healthcare workers [*n* (%)].

Section A. Knowledge domains/items	Correct	Incorrect	Do not know	Accuracy (%)	95% CI
Basic health concepts					
Complete understanding of health concept	5,203 (95.48)	189 (3.47)	57 (1.05)	95.48	94.85–96.11
Cognition of adolescent depression	4,967 (91.15)	378 (6.94)	104 (1.91)	91.15	90.34–91.96
Cognition of health check-up importance	4,821 (88.47)	502 (9.21)	126 (2.31)	88.47	87.51–89.43
Health knowledge					
Hepatitis B transmission routes	5,067 (92.99)	294 (5.39)	88 (1.61)	92.99	92.25–93.73
Tuberculosis treatment cognition	4,934 (90.55)	425 (7.80)	90 (1.65)	90.55	89.68–91.42
Blood pressure self-monitoring cognition	4,756 (87.28)	567 (10.41)	126 (2.31)	87.28	86.25–88.31
Infectious disease prevention cognition	5,156 (94.62)	223 (4.09)	70 (1.28)	94.62	94.01–95.23
Prenatal check-up cognition	4,287 (78.67)	892 (16.37)	270 (4.95)	78.67	77.46–79.88
Health skills					
BMI calculation method	4,978 (91.35)	345 (6.33)	126 (2.31)	91.35	90.53–92.17
Weight status judgment	4,823 (88.51)	456 (8.37)	170 (3.12)	88.51	87.56–89.46
Weight control methods	4,634 (85.04)	634 (11.64)	181 (3.32)	85.04	83.94–86.14
Disease risk assessment	4,756 (87.28)	567 (10.41)	126 (2.31)	87.28	86.25–88.31

Section B. Complex health skills	Full mastery	Basic mastery	Partial mastery	No mastery	*Mastery rate (%) **
Public health service cognition	4,623 (84.84)	658 (12.08)	125 (2.29)	43 (0.79)	96.92
Mental health promotion methods	4,789 (87.88)	523 (9.60)	98 (1.80)	39 (0.72)	97.48
Osteoporosis prevention measures	4,567 (83.81)	721 (13.23)	134 (2.46)	27 (0.50)	96.96
CPR first-aid skill	5,034 (92.39)	356 (6.53)	47 (0.86)	12 (0.22)	98.92
Inpatient treatment cognition	4,456 (81.77)	834 (15.30)	132 (2.42)	27 (0.50)	97.07

Accuracy (%) in Section A = Correct/Total × 100.

Mastery rate (%) in Section B = (Full mastery + Basic mastery)/Total × 100.

Totals are based on *n* = 5,449.

### 3.3 Status of health communication practice behaviors

Health communication behaviors among PHCWs included formal health education, digital media promotion, interpersonal education, and academic dissemination. As shown in Table 3, participation was highest in health education during consultations (98.73%), followed by health advice in daily life (97.50%), while engagement in academic dissemination remained relatively low (49.8%).

**Table 3:** Distribution of health communication practice behaviors among primary healthcare workers [*n* (%)].

Communication behavior type	Never/0 times	Occasional/1–2 times	Usually/3–5 times	Frequent/6–10 times	Always/>10 times	Participation rate (%)
Health science lectures	1,162 (21.33)	2,156 (39.57)	1834 (33.66)	1,023 (18.77)	436 (8.00)	78.67
Social media health promotion	1,623 (29.78)	1967 (36.10)	1,456 (26.72)	834 (15.30)	569 (10.44)	70.22
Health education during consultations	69 (1.27)	423 (7.76)	2,156 (39.57)	1967 (36.10)	834 (15.30)	98.73
Health advice in daily life	136 (2.50)	623 (11.43)	2067 (37.95)	1834 (33.66)	789 (14.48)	97.50
Academic exchange & publication	2,734 (50.18)	1834 (33.66)	756 (13.87)	98 (1.80)	27 (0.50)	49.82

### 3.4 Health knowledge acquisition and updating capacity

At the continuing medical education (CME) level, 4,287 (78.67%) participants reported involvement in at least one CME activity within the past year. Attendance at academic conferences was most frequent (*n* = 2,934, 53.84%), followed by exchange visits (*n* = 2,156, 39.57%), whereas participation in in-service degree programs remained relatively limited (*n* = 1,456, 26.72%) (Table 4).

**Table 4:** Participation in continuing education and knowledge update behaviors among primary healthcare workers [*n* (%)].

Education type/learning behavior	Participants	Participation rate (%)	Avg. participation (times)	95% CI
Continuing medical education				
Academic conferences	2,934 (53.84)	53.84	2.67 ± 1.45	2.62–2.72
Exchange visits	2,156 (39.57)	39.57	1.89 ± 1.23	1.84–1.94
In-service degree education	1,456 (26.72)	26.72	0.78 ± 0.89	0.73–0.83
Other forms	789 (14.48)	14.48	0.45 ± 0.67	0.42–0.48
Professional material learning				
Professional book reading (monthly)	4,690 (86.07)	86.07	4.23 ± 3.67	4.13–4.33
Literature review (monthly)	4,426 (81.23)	81.23	3.45 ± 3.23	3.36–3.54
Online resource utilization				
Dingxiang Yuan	3,267 (59.96)	59.96	–	–
WHO official website	2,834 (52.01)	52.01	–	–
Int’l society of hypertension	2,156 (39.57)	39.57	–	–
Other medical websites	1,623 (29.78)	29.78	–	–

Pearson correlation for normally distributed variables; Spearman correlation for non-normal variables.

In terms of independent professional learning, most healthcare workers reported regular engagement with professional materials. Specifically, 86.07% had read professional books or clinical guidelines at least once per month, and 81.23% had reviewed domestic or international scientific literature. However, the frequency varied, with only 19.99% reading such materials more than six times per month.

Regarding problem-solving strategies, the majority of respondents adopted evidence-based approaches when facing clinical or communication-related challenges. A large proportion reported consulting the literature (84.84%) and professional reference books (77.71%), while others sought advice from colleagues or domain experts (72.80%) or browsed professional websites (63.43%). These patterns indicate a generally proactive and self-directed attitude toward continuous professional development among PHCWs.

### 3.5 Influence of demographic and occupational characteristics on health communication competence

Univariate analysis demonstrated that both demographic and occupational factors were significantly associated with variations in health communication competence among primary healthcare workers (Table 5).

**Table 5:** Influence of demographic and occupational characteristics on health communication competence among primary healthcare workers (*n* = 5,449).

Variable	*N* (%)	mean ± SD	*t*/*F*	*P*-value
Gender			3.276	<0.001
Male	1750 (32.13)	67.92 ± 16.45		
Female	3,699 (67.87)	71.56 ± 15.72		
Age (years)				
≤30	908 (16.67)	69.34 ± 15.43	6.87	< 0.001
31–40	799 (14.66)	70.25 ± 16.02		
41–50	2,447 (44.91)	71.28 ± 15.48		
≥51	1,295 (23.76)	68.23 ± 16.92		
Education level			18.34	<0.001
Associate degree or below	2,330 (42.75)	65.12 ± 17.46		
Bachelor’s degree	3,100 (56.88)	71.82 ± 15.01		
Master’s degree or above	19 (0.35)	77.15 ± 12.64		
Profession			15.42	<0.001
Doctor	2,875 (52.80)	73.28 ± 14.35		
Nurse	1,310 (24.07)	68.21 ± 16.24		
Pharmacist	276 (5.07)	69.43 ± 15.61		
Technician	386 (7.09)	65.67 ± 17.83		
Others	602 (11.05)	66.91 ± 17.12		
Professional title			21.87	<0.001
None	1988 (36.48)	62.91 ± 18.12		
Junior title	1,534 (28.15)	69.12 ± 16.08		
Intermediate title	1,106 (20.28)	73.27 ± 14.46		
Associate senior title	712 (13.10)	78.04 ± 12.27		
Senior title	109 (2.01)	81.78 ± 10.14		
Type of Institution				
Township-level (township health centers, village clinics)	4,199 (77.06)	68.12 ± 16.25	9.02	<0.001
Community-level (community health centers/stations, subdistrict health centers)	960 (17.62)	71.05 ± 15.47		
Private/social-run (outpatient departments, clinics)	290 (5.32)	67.38 ± 17.08		
Work experience (years)				
<5	1,456 (26.72)	67.01 ± 17.62	9.48	<0.001
5–10	1834 (33.66)	69.83 ± 15.48		
10–20	1,623 (29.78)	74.21 ± 13.62		
≥20	536 (9.84)	68.32 ± 16.08		
Weekly working hours (h/week)				
≤30	306 (5.61)	68.54 ± 16.51	4.43	0.004
31–40	866 (15.89)	69.42 ± 15.72		
41–50	2,173 (39.85)	70.51 ± 15.11		
>50	2,104 (38.65)	69.78 ± 16.54		
Monthly income (CNY)				
<5,000	3,231 (59.32)	64.75 ± 18.16	13.52	<0.001
5,000–6,999	1,018 (18.68)	68.24 ± 16.08		
7,000–9,999	927 (17.02)	71.83 ± 14.43		
≥10,000	273 (5.02)	75.42 ± 13.11		

#### 3.5.1 Gender and age

Female healthcare workers achieved significantly higher communication competence scores (70.54 ± 15.62) than males (68.12 ± 16.78; *t* = 3.276, *P* < 0.001). Significant differences were also observed across age groups (*F* = 6.87, *P* < 0.001). Workers aged 41–50 years exhibited the highest average score (71.28 ± 15.48), followed by those aged 31–40 years (70.25 ± 16.02), while those aged ≤30 and ≥51 years scored relatively lower.

#### 3.5.2 Educational level and professional title

Health communication competence increased progressively with educational attainment (*F* = 18.34, *P* < 0.001). Participants holding bachelor’s degrees or higher demonstrated substantially greater competence (73.24 ± 14.29 for bachelor’s; 75.65 ± 13.24 for master’s or above) compared with those with junior college or secondary education. Similarly, higher professional title levels were associated with stronger communication competence (*F* = 21.87, *P* < 0.001), with senior-title workers achieving the highest mean score (75.42 ± 13.78).

#### 3.5.3 Institution type and work experience

Significant disparities were identified across healthcare institution types (*F* = 9.02, *P* < 0.001). Workers from community-level institutions (71.05 ± 15.47) demonstrated the highest communication competence, whereas those from township-level institutions scored lower (68.12 ± 16.25). Competence also varied significantly by years of work experience (*F* = 9.48, *P* < 0.001), showing an inverted U-shaped relationship—performance peaked among workers with 10–20 years of experience (74.21 ± 13.62).

#### 3.5.4 Working hours and income

Weekly working hours exerted a modest yet significant effect (*F* = 4.43, *P* = 0.004); those working 41–50 h per week achieved slightly higher competence scores (70.51 ± 15.11). Monthly income was also positively associated with competence (*F* = 13.52, *P* < 0.001): workers earning ≥¥10,000 per month scored the highest (75.42 ± 13.11), while those earning <¥5,000 scored the lowest (64.75 ± 18.16).

#### 3.5.5 Influence of health status and occupational stress on health communication competence

Analysis of individual well-being factors revealed that both self-rated health status and occupational stress significantly affected health communication competence among primary healthcare workers (Table 6). Participants with higher self-rated health reported markedly stronger competence (73.45 ± 14.23 for ≥80 points) compared with those with poorer health (<60 points: 61.78 ± 19.45; *t* = 8.67, *P* < 0.001). Similarly, occupational stress demonstrated a clear negative association with communication competence (*F* = 15.67, *P* < 0.001). Workers in the low-stress group achieved higher scores (72.34 ± 14.56) than those in the high-stress group (65.23 ± 17.89).

**Table 6:** Influence of health status and occupational stress on health communication competence among primary healthcare workers (*n* = 5,449).

Variable	*n* (%)	Communication competence score (mean ± SD)	*t*/*F*	*P*-value
Self-rated health status				
≥80 points	3,290 (60.38)	73.45 ± 14.23	8.67	<0.001
60–79 points	1731 (31.76)	67.89 ± 16.45		
< 60 points	428 (7.85)	61.78 ± 19.45		
Occupational stress level				
Low	1834 (33.66)	72.34 ± 14.56	15.67	<0.001
Medium	2,723 (49.97)	69.78 ± 15.89		
High	892 (16.37)	65.23 ± 17.89		

### 3.6 Multivariable analysis of influencing factors on health communication competence

Multiple linear regression analysis was employed to identify independent factors influencing health communication competence. The health communication competence score served as the dependent variable, and all variables with *P* < 0.05 in the univariate analysis were included as independent variables.

The results identified educational level (*β* = 0.239, *P* < 0.001), professional title (*β* = 0.192, *P* < 0.001), self-rated health status (*β* = 0.183, *P* < 0.001), occupational stress level (*β* = −0.151, *P* < 0.001), type of healthcare institution (*β* = 0.129, *P* < 0.001), and gender (*β* = 0.096, *P* = 0.002) as independent influencing factors (Table 7).

**Table 7:** Multiple linear regression analysis of factors influencing health communication competence among primary healthcare workers.

Variable	*B* (unstandardized coefficient)	SE	Standardized *β*	*t*-value	*P*-value	95% CI for *B*
Education level	4.128	0.553	0.239	7.465	<0.001	3.044–5.212
Professional title	3.674	0.621	0.192	5.914	<0.001	2.456–4.892
Health status	0.182	0.033	0.183	5.515	<0.001	0.118–0.246
Occupational stress	−2.764	0.441	−0.151	−6.268	<0.001	−3.629 – –1.899
Institution type	2.018	0.417	0.129	4.838	<0.001	1.201–2.835
Gender	1.745	0.563	0.096	3.099	0.002	0.642–2.848
Age	0.854	0.336	0.066	2.54	0.011	0.194–1.514
Monthly income	1.189	0.448	0.076	2.655	0.008	0.310–2.068
Work experience	0.546	0.229	0.054	2.384	0.017	0.098–0.994

*B* represents unstandardized regression coefficients; *β* represents standardized coefficients. CI, confidence interval.

The overall model demonstrated good fit (*R*^2^ = 0.421, Adjusted *R*^2^ = 0.410, *F* = 88.72, *P* < 0.001), indicating that the included variables explained 42.1% of the variance in health communication competence. Standardized regression coefficients revealed that educational level had the strongest positive effect, followed by professional title and self-rated health status, while occupational stress remained a significant negative predictor.

Although working conditions were discussed in the narrative interpretation, they were represented in the model through related variables—specifically, type of institution, weekly working hours, and occupational stress—and thus were not treated as a separate factor in the regression analysis.

**Figure 1:**
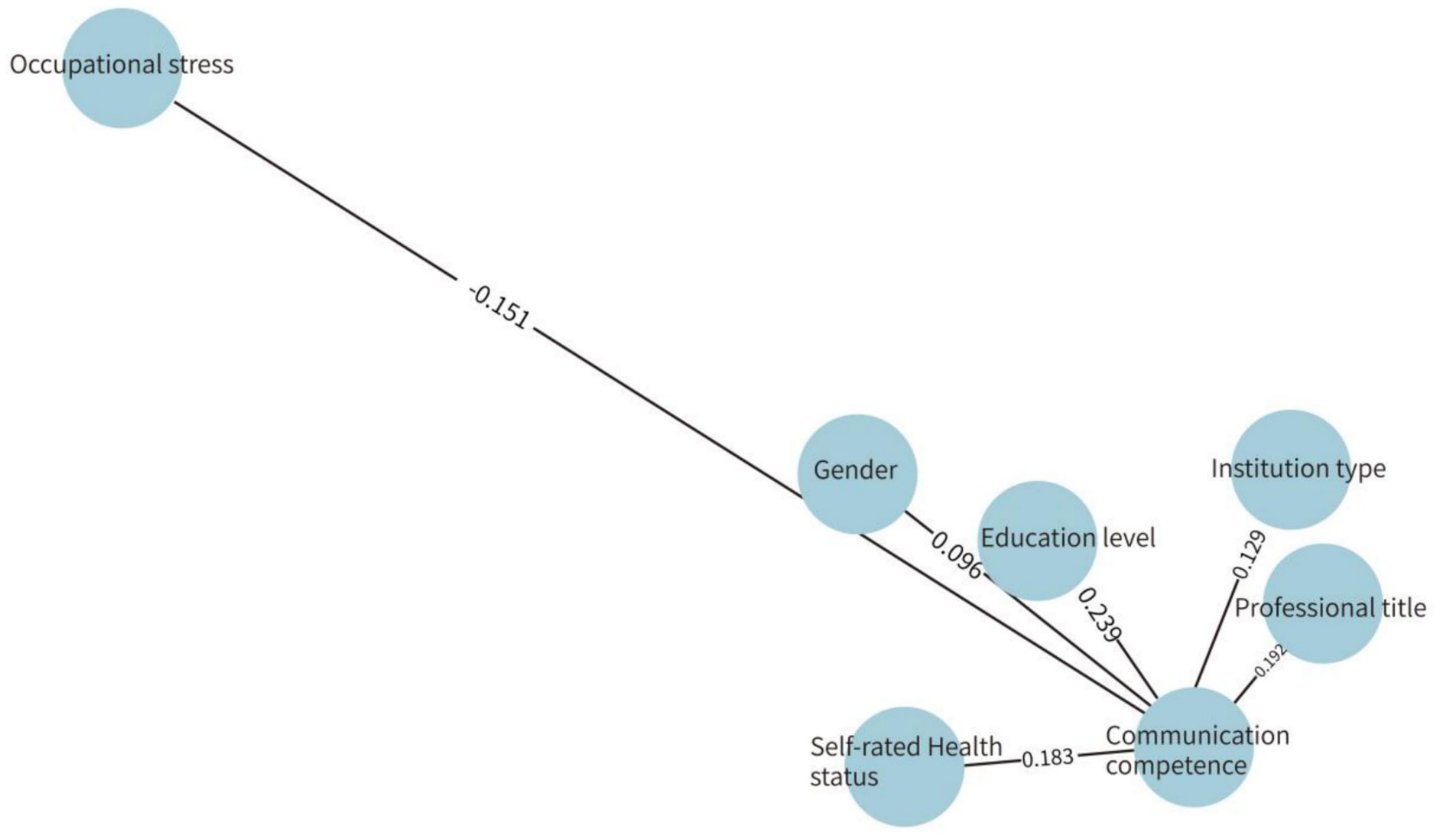
Structural equation model of factors influencing health communication competence among primary healthcare workers. Model fit indices: *χ*^2^/df = 2.34, RMSEA = 0.045, CFI = 0.96, TLI = 0.95.

Pathway analysis results suggested that individual characteristic factors (education, title) may be associated with communication competence through professional competence and learning ability, while occupational environmental factors (e.g., institution type and workload-related conditions) relate to communication competence via work opportunities and access to resources. Moreover, self-rated health status and occupational stress were correlated with communication performance. This model provides a theoretical basis and identifies intervention targets for enhancing the health communication competence of primary healthcare workers, as summarized in Table 8 (see Figure 1).

**Table 8:** Pathway analysis results of factors influencing health communication competence among primary healthcare workers.

Influence pathway	Direct effect	Indirect effect	Total effect	Mediation proportion (%)	*P*-value
Education level → communication competence	0.239	0.087	0.326	26.69	<0.001
Professional title → communication competence	0.192	0.065	0.257	25.29	<0.001
Self-rated health status → communication competence	0.183	0.033	0.216	15.28	<0.001
Occupational stress → communication competence	−0.151	−0.043	−0.194	22.16	<0.001
Institution type → communication competence	0.129	0.054	0.183	29.51	<0.001
Gender → communication competence	0.096	0.022	0.118	19.04	0.002

SEM further validated the direct and indirect effects of these factors on health communication competence. The updated model, incorporating revised standardized coefficients, exhibited a good fit (*χ*^2^/df = 2.34, RMSEA = 0.045, CFI = 0.96, TLI = 0.95). All indices met the recommended standards (*χ*^2^/df < 3, RMSEA < 0.08, CFI > 0.90, TLI > 0.90), indicating satisfactory model adequacy (see Figure 1).

## 4 Discussion

### 4.1 Overall moderate level of health communication competence with notable knowledge-practice gap

The comprehensive assessment conducted in this study indicates that the health communication competence of PHCWs in China remains at a moderate level, with a mean score of 69.45. This result is particularly significant considering the strategic importance of primary care within the Healthy China 2030 framework. The distribution of competence levels, with only 20.04% of PHCWs achieving high proficiency, underscores a substantial deficiency that may hinder the effective delivery of preventive services and health education at the community level. This pattern is consistent with international evidence suggesting that health promotion competencies are often under-developed in primary care settings, especially in resource-constrained environments (8).

A key finding of this study is the discrepancy between theoretical knowledge and practical application. Although PHCWs exhibited relatively high levels of health literacy knowledge (78.32), their scores for communication practice behavior were considerably lower (62.78). This pronounced knowledge–practice gap implies the presence of structural and organizational barriers that obstruct the translation of acquired knowledge into effective patient communication and community engagement. The persistence of this gap may stem from entrenched institutional priorities that emphasize curative rather than preventive care, resulting in insufficient time, incentives, and resources for health education initiatives (9). Additionally, many PHCWs lack formal training in health communication techniques, particularly in areas such as audience analysis, message adaptation, and culturally appropriate communication. These competencies are essential for bridging the divide between professional expertise and public understanding (10).

In addition to the overall patterns of competence, the findings related to learning behaviors and problem-solving strategies further highlight the proactive but uneven professional development characteristics of primary healthcare workers. The majority demonstrated strong initiative in acquiring new knowledge, as evidenced by frequent reading of professional books and clinical guidelines as well as regular engagement with Chinese and English literature. Notably, when facing clinical or communication-related challenges, most respondents adopted evidence-based problem-solving approaches. A substantial proportion reported consulting scientific literature (84.84%) and professional reference books (77.71%), while many also sought advice from colleagues or domain experts (72.80%) or accessed reputable online medical resources (63.43%).

These patterns indicate that PHCWs generally possess adequate motivation and learning habits to support continuous professional growth. However, the reliance on self-directed learning also reflects potential structural constraints—including limited access to formal, standardized training and the absence of systematic communication-skills curricula— which may contribute to the persistent knowledge-practice gap observed in this study. Strengthening institutional support mechanisms, such as provision of structured communication training, mentorship systems, and high-quality learning platforms, may help ensure that the strong individual initiative exhibited by PHCWs can be effectively translated into improved communication practices.

### 4.2 Educational attainment as the primary determinant of health communication competence

Multivariable analysis identified educational level as the strongest predictor of health communication competence (*β* = 0.245), highlighting the crucial role of formal education in shaping the cognitive and analytical capacities necessary for effective communication in healthcare settings. Higher levels of education are generally associated with improved information-processing abilities, critical thinking skills, and a stronger orientation toward evidence-based practice. These attributes are essential for accurately assessing patients’ informational needs, synthesizing health knowledge, and conveying messages clearly and credibly (11). The positive relationship between educational attainment and communication competence is consistent with established health communication frameworks, which emphasize that the effectiveness of information exchange depends on both content knowledge and procedural knowledge—that is, knowing what to communicate and how to communicate it effectively (12). This finding underscores the importance of strengthening educational pathways for primary healthcare workers, including opportunities for continuing professional education and formal training in communication and health promotion. Enhancing educational access and curricula focused on communication competence may therefore serve as a strategic intervention to improve the quality of primary healthcare delivery and public health education. Gender differences in health communication competence may partly reflect gender-related communication norms and role expectations reported in prior studies, which suggest that female healthcare workers often demonstrate greater engagement in patient-centered communication (13). This finding suggests potential benefits from incorporating communication styles typically associated with female socialization into training programs for all PHCWs.

### 4.3 Occupational and work-related context as determinants of communication competence

The significant differences in communication competence across various types of primary healthcare institutions highlight the influence of occupational and work-related contexts. Primary healthcare workers employed in community-level institutions demonstrated higher competence scores than those in township or village facilities. This disparity reflects the effect of institutional type, professional exposure, and access to resources such as training opportunities and interdisciplinary collaboration (14). The observed pattern parallels broader urban–rural inequalities in healthcare resource distribution across China and underscores that efforts to strengthen health communication must also address these contextual disparities in support and infrastructure (15).

The inverted U-shaped association between work experience and communication competence, with peak performance among individuals with 10–20 years of experience, suggests that communication skills develop progressively through clinical practice and accumulated interactions. However, the subsequent decline among more senior workers may be related to factors such as work fatigue, limited continuing education, or reduced motivation. This finding reinforces the importance of sustained professional development throughout the career trajectory rather than concentrating training efforts exclusively on early-career staff (16).

Furthermore, the positive association between income level and communication competence indicates that economic incentives and job satisfaction may contribute to improved engagement and motivation to apply effective communication behaviors. Collectively, these results demonstrate that contextual work-related factors—rather than purely individual attributes—play an essential role in shaping the communication capacity of primary healthcare workers.

### 4.4 Self-rated health status and occupational stress as independent influencing factors

This study identified both self-rated health status and occupational stress as significant predictors of health communication competence among primary healthcare workers. The positive association between self-rated health and communication performance suggests that better physical and psychological well-being enhances the cognitive and emotional capacity required for effective communication (17). Healthcare workers who report good health are more likely to demonstrate higher energy levels, emotional stability, and motivation, all of which facilitate active engagement in patient interactions and community outreach activities. Conversely, poorer self-rated health may limit attentional focus and interpersonal responsiveness, leading to reduced communication efficacy (18).

Occupational stress was found to exert a significant negative effect on communication competence (*β* = −0.151), indicating that sustained psychological strain can undermine both the quality and consistency of communication practices. Prolonged exposure to stress may impair emotional regulation, reduce empathy, and restrict the time and cognitive resources available for patient-centered dialogue (19). In addition, individuals experiencing high levels of stress may have less capacity or motivation to participate in continuing education, further perpetuating deficits in communication skills (20).

The presence of both direct and indirect associations between occupational stress and communication competence underscores the importance of integrating stress management and well-being interventions into professional development programs. Promoting mental health resilience and reducing work-related stressors may therefore serve as effective strategies for improving communication competence and, by extension, the quality of primary healthcare delivery.

### 4.5 Implications for multilevel intervention strategies

The complex interaction among individual, organizational, and systemic factors identified in this study underscores the need for comprehensive, multilevel intervention strategies.

At the individual level, training programs should extend beyond the transmission of theoretical knowledge to include the development of practical communication competencies. The content of such programs should be tailored to the educational background, professional role, and career stage of PHCWs to ensure relevance and effectiveness (21).

At the organizational level, healthcare institutions should foster supportive environments that emphasize health communication as a core professional responsibility. This may include adjusting workloads, establishing performance-based incentives, and allocating dedicated time for patient education activities (22).

Reducing urban–rural disparities in communication competence requires targeted investment in rural healthcare infrastructure. Enhancing digital connectivity can facilitate access to continuing education and professional development resources (23). The implementation of “Internet + Health Communication” training models could further benefit PHCWs in remote areas by providing equitable access to learning opportunities and expert guidance regardless of geographic constraints (24).

Finally, interventions aimed at improving the health and well-being of PHCWs—such as mental health support services, stress management initiatives, and the promotion of balanced workload expectations—are essential for sustaining communication competence and preventing professional burnout over time (25).

### 4.6 Limitations

The limitations should be acknowledged. (1) Causality and study design: this study employed a cross-sectional design, which limits the ability to establish causal relationships among variables. Although SEM was used to explore potential pathways among education level, health knowledge, stress, and communication competence, the identified associations should be interpreted as correlations rather than confirmed cause–effect links. For example, while the model suggests that education and work experience may contribute to improved health communication competence, it is also possible that individuals with higher competence are more likely to pursue further education or professional development. Similarly, the relationship between stress and competence may be bidirectional, in which high stress could impair communication competence, or conversely, lower competence could increase occupational stress. Longitudinal or experimental studies would be necessary to clarify the temporal and causal directions of these relationships. (2) Generalizability of findings: the present study was conducted exclusively among PHCWs in Zibo City, Shandong Province, which may limit the generalizability of the findings to other regions of China. Differences in socioeconomic development, healthcare resources, and local policy implementation may influence PHCWs’ communication competence and related factors in other provinces. No region-specific contextual factors, such as health policies or management structures, were analyzed in this study, which may affect the applicability of the results to broader contexts. Furthermore, the exclusion of PHCWs with less than 6 months of work experience may have introduced selection bias by favoring individuals who have already adapted to their professional roles and potentially possess higher communication competence. Future studies should include multi-provincial samples and PHCWs with varying lengths of service to enhance representativeness and external validity. (3) Potential biases associated with data collection: as this study relied on self-reported questionnaires, the findings may be affected by social desirability bias, particularly in items related to perceived communication competence and occupational stress. Although the survey was conducted anonymously, no additional measures were applied to control for this bias, which might have led to overestimation of competence or underreporting of stress. In addition, while the Occupational Stress Inventory–Revised (OSI-R) has been validated in healthcare settings, other adapted tools used in this study—such as the Health Communication Competence and Health Literacy Assessment Scales—have not yet been extensively validated among Chinese PHCWs. These factors may influence the accuracy and representativeness of the results. Future studies should combine self-reported data with objective indicators or supervisor assessments to strengthen reliability.

## 5 Conclusion

This study provides a comprehensive assessment of health communication competence among PHCWs in Zibo City, Shandong Province. The overall level of competence was found to be moderate, characterized by a clear knowledge–practice gap between theoretical understanding and practical communication behavior.

Multivariable analysis identified educational attainment, professional title, type of institution, work experience, self-rated health status, and occupational stress as significant factors influencing communication competence. Among these, educational attainment emerged as the primary determinant, emphasizing the crucial role of formal education and continuous professional learning in enhancing communication capacity. The study also highlights the need to address disparities across different institutional contexts and to improve the working and learning environments of PHCWs. Interventions aimed at promoting mental well-being, managing occupational stress, and expanding access to structured communication training are essential for sustaining competence development. While the findings offer valuable guidance for policymakers and healthcare administrators, they should be interpreted with caution due to the study’s cross-sectional design and its focus on a single geographic area. Future research involving multi-center and longitudinal studies across diverse regions of China is recommended to validate and extend these conclusions.

## Data Availability

The raw data supporting the conclusions of this article will be made available by the authors, without undue reservation.
