# Correction: A multicenter cross-sectional survey on the status and influencing factors of health communication competence among primary healthcare workers

**DOI:** 10.3389/fpubh.2026.1807296

**Published:** 2026-06-05

**Authors:** Xian Zhao, Jialin Zhao, Zhixia Shi, Tingting Li, Lili Zhao, Rou Li, Ling Li

**Affiliations:** 1 Institute of Health Education, Zibo Center for Disease Control and Prevention, Zibo, Shandong, China; 2 Department of Maternal and Child Health, Zichuan District Hongshan Health Center, Zibo, Shandong, China; 3 Department of Food Nutrition and Student Health, Zibo City Zhangdian District Disease Control and Prevention Center, Zibo, Shandong, China

**Keywords:** primary healthcare workers, health communication, health literacy, influencing factors, cross-sectional study

Specifically, the **Methods** section of the abstract on Page 1 and the first sentence of Section 2.1 both mention that data collection was conducted in August 2023. While this research project was indeed launched in 2023 and finalized in October 2025, its initial design centered on health communication content within medical and health institutions.

During the pre-defense in July 2025, the review experts put forward a critical suggestion to supplement data from medical staff. The authors attached great importance to this recommendation and completed the supplementary survey and analysis of medical staff data in August 2025. This supplementary work greatly enhanced the data support and conclusions of the study, which enabled the project to successfully pass the final defense in October 2025.

It should be emphasized that the core analysis of this paper is based on the final and complete dataset obtained from this supplementary survey. To uphold the academic rigor of the paper, the two aforementioned time references are changed from 2023 to 2025.

A correction has been made to the section **Abstract**, Methods:

“**Methods:** A cross-sectional survey was conducted in August 2025 among 5,449 primary healthcare workers in Zibo City, Shandong Province, using multi-stage stratified random sampling. A self-designed questionnaire assessed four dimensions: health literacy mastery, knowledge acquisition ability, communication practice behavior, and policy cognition level. Statistical analyses included t-tests, ANOVA, multiple linear regression, and structural equation modeling (SEM).”

A correction has been made to the section **2. Methods**, 2.1.Study Population and Sampling:

“A multi-center, cross-sectional survey was conducted in Zibo City, Shandong Province, in August 2025. Participants were selected using a multi-stage stratified random sampling method. Primary healthcare institutions were first stratified by type (community health service centers/stations, township health centers, village clinics, outpatient departments, and private clinics). Within each stratum, institutions were randomly selected using a random number table.”

A correction has been made to the section, **Funding:**

“The author(s) declared that financial support was received for this work and/or its publication. 2022 Shandong Province Humanities and Social Sciences Project, Project Number: 2022-ZXJK-13. The study was funded in 2022 and implemented in 2023, with subsequent data analysis and manuscript preparation completed in 2025.”

The original version of this article has been updated.

